# Peanut Allergen Threshold Study (PATS): validation of eliciting doses using a novel single-dose challenge protocol

**DOI:** 10.1186/1710-1492-9-35

**Published:** 2013-09-12

**Authors:** Giovanni A Zurzolo, Katrina J Allen, Steve L Taylor, Wayne G Shreffler, Joseph L Baumert, Mimi L K Tang, Lyle C Gurrin, Michael L Mathai, Julie A Nordlee, Audrey DunnGalvin, Jonathan O’B Hourihane

**Affiliations:** 1 Murdoch Childrens Research Institute, Melbourne, Australia; 2 Biomedical and Lifestyle Diseases Unit, School of Biomedical and Health Sciences, Victoria University, Melbourne, Australia; 3 Department of Allergy and Immunology, Royal Children’s Hospital, Melbourne, Australia; 4 University of Melbourne Department of Paediatrics, Melbourne, Australia; 5 Food Allergy Research and Resource Program, University of Nebraska, Lincoln, Nebraska; 6 Food Allergy Centre and the Centre for Immunology and Inflammatory Disease, Massachusetts General Hospital/Harvard Medical School, Boston, USA; 7 Melbourne School of Population and Global Health, University of Melbourne, Melbourne, Australia; 8 Paediatrics and Child Health, University College, Cork, Ireland

**Keywords:** Eliciting dose (ED), Food Allergy related Quality of Life Questionnaires-(FAQLQ), Single dose, Peanut thresholds, Oral Food Challenges (OFC), Voluntary Incidental Trace Allergen Labelling (VITAL), Peanut Allergen Threshold Study (PATS)

## Abstract

**Background:**

The eliciting dose (ED) for a peanut allergic reaction in 5% of the peanut allergic population, the ED05, is 1.5 mg of peanut protein. This ED05 was derived from oral food challenges (OFC) that use graded, incremental doses administered at fixed time intervals. Individual patients’ threshold doses were used to generate population dose-distribution curves using probability distributions from which the ED05 was then determined. It is important to clinically validate that this dose is predictive of the allergenic response in a further unselected group of peanut-allergic individuals.

**Methods/Aims:**

This is a multi-centre study involving three national level referral and teaching centres. (Cork University Hospital, Ireland, Royal Children’s Hospital Melbourne, Australia and Massachusetts General Hospital, Boston, U.S.A.) The study is now in process and will continue to run until all centres have recruited 125 participates in each respective centre.

A total of 375 participants, aged 1–18 years will be recruited during routine Allergy appointments in the centres. The aim is to assess the precision of the predicted ED05 using a single dose (6 mg peanut = 1.5 mg of peanut protein) in the form of a cookie. Validated Food Allergy related Quality of Life Questionnaires-(FAQLQ) will be self-administered prior to OFC and 1 month after challenge to assess the impact of a single dose OFC on FAQL. Serological and cell based in vitro studies will be performed.

**Conclusion:**

The validation of the ED05 threshold for allergic reactions in peanut allergic subjects has potential value for public health measures. The single dose OFC, based upon the statistical dose-distribution analysis of past challenge trials, promises an efficient approach to identify the most highly sensitive patients within any given food-allergic population.

## Introduction

**Figure 1:**
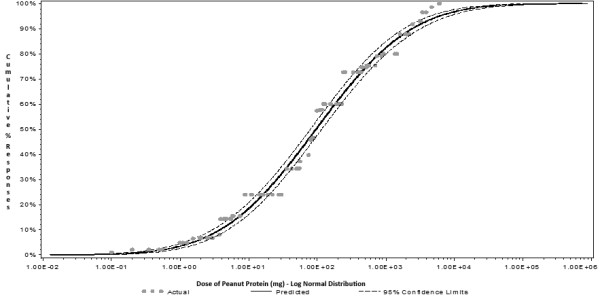
**Population dose distribution models for peanut thresholds.** Adapted from the manuscript title “Clinical challenge data for development of allergen management thresholds for precautionary labeling of foods- VITAL 2.0” [1].

The eliciting dose (ED) for a peanut allergic reaction in 5% of the peanut allergic population (ED05) has been estimated at 1.5 mg of peanut protein. This ED05 estimate was derived from the statistical dose- distribution of peanut allergic individuals (children and adults). All individuals participated in oral food challenge (OFC) protocols that use graded, incremental doses administered at short, fixed time intervals, as shown in Figure 1, with a strong, monotonic relationship between dose and the proportion of study participants reacting at each actual or extrapolated dose [1]. It is not always possible to determine whether a reaction has occurred to a *discrete* threshold dose of allergen or alternatively has been the result of the *cumulative* dose consumed by the allergic individual at the time of reaction. Statistical methods can be used to model the dose-distribution of the peanut-allergic population when the precise threshold dose is known to fall within a defined dosing interval but the exact threshold value is unknown [2,3]. Since the ED05 is derived from statistical dose-distribution models of the peanut-allergic population, it is important to clinically validate that this dose is predictive of the allergenic response in a further unselected group of peanut-allergic individuals.

This issue is of importance to all stakeholders in food allergy because over the last 10 years an increasing number of food manufacturers have incorporated voluntary allergen precautionary statements which advise the allergic consumer of the potential presence of allergens using “may contain allergen” statements which are not legislated for and are variable in content around the world [4]. Regulatory thresholds for allergen labelling currently do not exist in most countries, with the exception of Japan and Switzerland. Voluntary industry-led initiatives that use clinical thresholds as the basis for precautionary labelling decisions are based on ED estimates derived from multiple dosing food challenges. Although attempts to improve labelling have been introduced in some countries (e.g. Australia with Voluntary Incidental Trace Allergen Labelling VITAL 2.0), these are still hampered by being voluntary and currently are considered to lack credibility [5].

This study aims to assess the precision of the predicted ED05 using a single dose (6 mg peanut = 1.5 mg of peanut protein, approximately 1/100th of a peanut kernel) challenge and to validate the modelling that has been used to develop precautionary labelling criteria for VITAL 2.0, as currently VITAL 2.0 uses ED01 (0.2 mg of peanut protein) to estimate its reference doses [6]. In addition this study will examine whether 95% of peanut-allergic consumers are tolerant of an amount that is more than 5 times higher than the VITAL ED01 threshold, thus suggesting if 95% of participants are tolerant to an ED05 then there would be an exceedingly low probability that they would react to an ED01. The ED05 has been chosen pragmatically as it will allow the study to proceed with the recruitment of an achievable number of peanut-allergic individuals to provide sufficient statistical power to validate the accuracy of the population threshold distribution of peanut allergic individuals (discussed in detail below). A validation study of the ED01 would have required a prohibitively large, much more expensive study. In contrast it would be feasible to study further the 5% of subjects who DO react at ED05, with lower doses, including the ED01.

We feel it is important to standardise this approach at an international level since the findings in this study have consequences for the food manufacturing industry at a global level. Our plans to initiate this study have recently been supported in a review by a large multidisciplinary European group [7]. This may contribute to improvement of precautionary labelling thresholds to be set for use by regulators and manufacturers to protect the food allergic consumer.

## Methods

### Recruitment

This is a multi-centre study involving three teaching centres. A total 375 participants will be recruited (125 in each centre) during their follow-up appointments in the Department of Allergy in each respective centre.

### Inclusion criteria

Each patient must meet all of the following criteria to be enrolled in this study.

•Age between 1 to 18 years old and

•Demonstrate evidence of peanut allergy as defined by either

(a) History of unequivocal exposure (including accidental) and typical acute allergic reaction within the preceding 2 years and positive peanut SPT/sIgE, or

(b) Positive oral food challenge with peanut performed within 2 years - either open oral food challenge or DBPCFC (Double-blind, placebo-controlled food challenges)

(c) Peanut never ingested, but sensitisation to peanut above the 95% positive predictive value (PPV) for clinical allergy, i.e. peanut serum IgE ≥ to 15 kU/L (by CAP FEIA) and/or peanut SPT wheal size ≥ to 8 mm within 2 months of the single dose challenge.

### Exclusion criteria

Patients meeting any of the following criteria will be excluded from the study.

•Family or child does not consent to participate

•Medically unfit for challenge according to local unit OFC guidelines/protocol (e.g., high fever, unwell with intercurrent illness,

•Any objective sign of an acute allergic reaction

•Oral corticosteroids within 14 days prior to challenge

•Episode of anaphylaxis of any cause in 4 weeks prior to challenge

•Use of antihistamines within 5 days of oral food challenge

•Asthma that is not well controlled as demonstrated by FEVI < 85% of predicted best.

### Food Allergy related Quality of Life Questionnaires-(FAQLQ)

**Figure 2:**
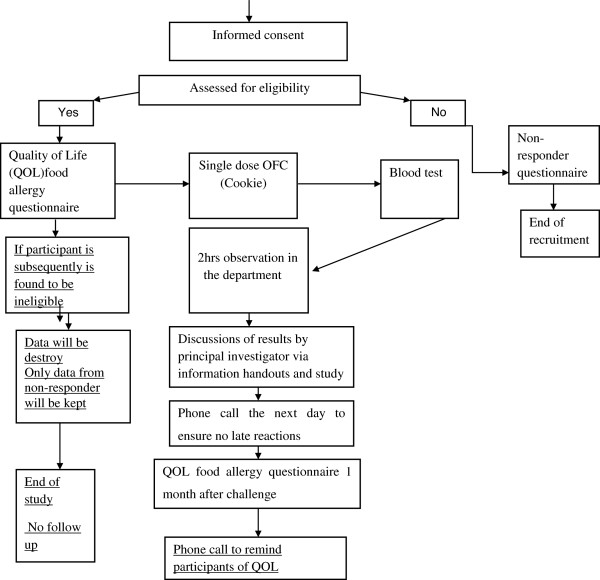
Study design diagram.

Validated FAQL questionnaires will be self-administered prior to OFC and 1 month after challenge to assess whether the impact of this novel single dose OFC protocol is similar to that of “routine” diagnostic OFC, (Figure 2) (Additional files 1, 2 and 3).

### Non-Responder Questionnaire (NRQ)

We aim to administer a non-responder questionnaire (NRQ): a set of questions intended to permit comparison of basic demographic and clinical allergy data in those choosing not to participate and in study participants (Additional file 4). The NRQ that we have developed is similar to the NRQ that was used by Osborne et al. (2010) [8].

### Single dose Oral Food Challenge (OFC)

A standard OFC administers multiple doses over 45–120 minutes depending on the challenge protocol. We will give a single dose of peanut, taken in isolation, at the level of the predicted ED05 (6 mg whole peanut = 1.5 mg peanut protein) in the form of a cookie consisting of granulated sugar, brown sugar, all-purpose wheat flour, vegetable shortening, salt and baking soda. Peanut flour will be added at a level that represents 6 mg whole peanut equivalent to 1/100th of whole peanut. For subjects allergic to other cookie ingredients e.g. wheat, the peanut dose will be administered in a food known to be tolerated. The challenge materials are shelf-stable and are manufactured at The University of Nebraska and then distributed to participating clinic centres.

### Criteria for a positive OFC result

Only objective criteria will be used in the validation of the ED05, since that dose was predicted on the basis of challenge-associated objective responses only. Objective criteria are outlined by Sampson et al. in the PRACTALL criteria [9] and have been validated in the Healthnuts study [10]. These criteria include urticaria, perioral or periorbital angioedema, vomiting, diarrhoea, respiratory or cardiovascular compromise (including anaphylaxis) and rhinoconjuctivitis. All objective signs will be quantitated in number, site and duration of presence. Participants in OFC often expect severe outcomes following ingestion; this may manifest as subjective symptoms. Subjective symptoms will be recorded but not used in the analysis of the reactions to validate the derived ED05 because the ED05 was developed only on the basis of objective reactions. Subjective symptoms to be recorded include: Headache, dizziness, bloating, abdominal pain, cramps, muscle aches, aching joints, anxiety, tension, agitation [11,12].

The prior agreed objective criteria for a positive OFC result are any objective signs occurring within 2 hours of ingestion. All objective signs will be recorded:

•3 or more concurrent noncontact urticaria persisting for at least 5 minutes;

•perioral or periorbital angioedema;

•rhinoconjunctivitis

•diarrhoea

•vomiting (excluding gag reflex); or

•evidence of circulatory or respiratory compromise (anaphylaxis eg, persistent cough, wheeze, change in voice, stridor, difficulty breathing, and collapse) [10].

### Blood test

A blood sample (10 ml) will be taken for peanut component analysis and quantitative peanut-specific IgE fluoroenzyme immunoassays 20 minutes after OFC.

### Sample size estimation

The population proportion of peanut allergic children who react to the nominal ED05 dose of peanut will be estimated, separately for each of the three participating centres, as the corresponding observed proportion of participants. If, based on these three proportions, there is strong evidence against the null hypothesis that the proportion reacting is the same in all three centres then centre-specific estimates will be reported, otherwise the proportion aggregated over all three centres will serve as a single centre-independent estimate. 95% confidence intervals for these population proportions will be calculated using the properties of the binomial distribution. Example of 95% confidence intervals for sample sizes 70, 100, 150, 200 and 375 if the estimated prevalence is equal to the nominal value of 5%, are displayed in Table 1. A sample size of 150 corresponds to a lower confidence limit of 2.3% and an upper confidence limit of 10%. While this implies that the population proportion may be as little as half or as much as double the observed proportion, this calculation is conservative since it uses the sample size expected in a single centre, not from the three centres combined, so it is sufficiently accurate to rule out gross incompatibility between the nominal and observed proportion of participants reacting.

**Table 1:** Projected 95% confidence intervals for the prevalence of clinical reactivity in peanut allergic children and adults receiving the ED_05_ dose (6 mg of whole peanut = 1.5 mg of peanut protein) for sample sizes ranging from 70 to 200

**Sample size (of peanut allergic individuals)**	**Value of target prevalence (5% for the ED_05_)**	**Projected 95% confidence interval**
70	5%	0.9% - 12%
100	5%	1.6% - 11%
150	5%	2.3% - 10%
200	5%	2.4% - 9%
375	5%	3.1% - 7.8%

Summary statistics will be used to compare the features of participants and non-participants, and of ED05-reactors and non-reactors. Variables to be examined will include clinical severity of previous reactions, age, sex, SPT wheal size and peanut component-specific IgE levels. Multivariable logistic regression analyses will be used to identify combinations of these features that identify the low-dose reactors.

### Ethics/Patient safety

This Study has been approved by Cork University Hospital Research Ethics Committee (ECM 4 g), Royal Children’s Hospital Human Research Ethics Committee (HRECApp 32166A), and Massachusetts General Hospital Research Ethics Committee (2012P002475). Written, informed parental and adolescent consent and assent from younger children will be recorded before participation in the PATS challenge. An External Safety Monitor has been appointed who is an experienced allergist, not otherwise involved in this study or related studies in the study centres.

## Discussion

The estimation of the threshold dose for allergic reaction to peanut in peanut allergic subjects has potential value for public health measures. The use of statistical dose-distribution modelling based upon the results of low-dose clinical challenges of peanut-allergic individuals has been viewed as a strong approach to estimation of the population threshold for peanut [13,14].

However, the clinical determination of individual thresholds is based upon graded incrementally increasing challenge doses administered at convenient time intervals, sometimes as short as 15–20 minutes between doses. The individual threshold doses are frequently reported as cumulative doses because it is impossible to claim that each dose is fully assimilated before administration of the next dose [15].

Allen et al. (2013) used this approach to estimate a population threshold for the peanut-allergic population based upon challenges of 750 individuals. The ED05 from the log normal dose-distribution was 6 mg of whole peanut or 1.5 mg of peanut protein. Since cumulative doses were used in the evaluation of individual challenges and subsequent statistical dose-distribution modelling, it is important to validate the peanut ED05 using a single-dose approach. Peanut is the best-studied food allergen in terms of low dose OFC to date. This novel PATS approach could be adapted for other major food allergens, if this proposed clinical study supports the statistically determined ED05 based upon population dose-distribution modelling [1].

The plan to approach all peanut allergic subjects in 3 distinct geographical regions the varied or permissive entry criteria and the analysis of the non-participants will address the most common criticism of OFC studies: how representative of the general peanut allergic population are the subjects who volunteered? Peanut allergic subjects who have food challenges are highly selected and they may not represent the whole spectrum of reactivity to peanut in peanut allergic subjects [16].

The strict requirement for only objective signs being used to determine a case is important, because subjective reactions are known to resolve during a routine OFC that is continued until objective signs are recorded [10,17].

Peanut allergic patients are usually advised to avoid foods that are labelled as “may contain” peanut. A recent study by Madsen et al. (2012) has showed that it is understood and accepted by clinicians, patients and food producers that zero risk is not a realistic or attainable option [18]. However clinical risk communications that are not specific may increase anxiety and risk taking behaviours without increasing awareness, confidence or safety [7].

Currently there is no standard approach being used by all manufacturers in relation to precautionary labelling. This may be due, in part, to the lack of agreement among the scientific community regarding clinically safe threshold levels. If this current study validates the ED05 this will aid the scientific and medical communities and also the manufacturing industry in the use of quantitative precautionary labelling, backed with sound scientific evidence for the establishment of safe threshold levels for 95% of the peanut allergic community.

The PATS study offers a new clinical paradigm and methodology with regards to assessing clinical risk; this current study may potentially define the 5% of patients who are most highly sensitive. Validated questionnaires assessing FAQL have shown patients gain nearly as much from a “failed” OFC as they do from a “passed” OFC, probably due to decreased uncertainty about the next and future reactions [19] and we hypothesise that individual families may also show such an improvement after a PATS single dose challenge. This tangible impact could promote adoption of PATS single dose peanut challenges in units not currently performing diagnostic OFC. If this proposed clinical study supports the statistically determined ED05 based upon population dose-distribution modelling of peanut, it may show promise for clinical validation of other allergenic food sources where sufficient threshold data is available to model the population dose-distribution. Eventually a single-dose diagnostic OFC using other food allergens may be adopted as well.

Clinicians may be able to use PATS single dose OFCs as they are easier to perform than routine diagnostic OFC or DBPCFC and they could contribute to the complex analysis of risk that clinicians currently make in a heuristic fashion that varies between practitioners. Currently clinicians make value judgements about whether they believe a child to be exquisitely sensitive to a food or not and therefore what to advise with regards to avoiding trace amounts of allergen in food (i.e. foods with precautionary labelling).

The single dose protocol does not replace current clinical food challenges which are for the diagnosis of food allergy but would provide extra clinical information of patients’ level of risk and could help inform consumer choices and physician advice to patients regarding precautionary labelling [20,21]. This project may offer a practical way to discern whether allergic patients can safely ingest foods with labels such as “may contain traces”, although this outcome would require collaboration with the food industry and more uniform adoption of criteria for use of precautionary labels as proposed in the Australian VITAL strategy.

## Conclusion

The PATS single dose OFC, based upon the statistical dose-distribution analysis of past challenge trials, promises an efficient approach to identify the most highly sensitive patients within any given food-allergic population. The peanut protocol described herein will evaluate the practicality of this approach and allow assessment of its safety. The validation of the ED05 originally statistically determined from the dose-distribution analysis would be a major benefit of the study as it would serve to inform governments in the application of a more transparent and sensible approach in the use of precautionary labelling. It will also aid public health agencies in the establishment of approaches to allergen management that will protect the vast majority of food-allergic consumers/patients.

## Competing interests

GZ declares that he has no competing interests. JH has received speaker honoraria and travel support from Stallergenes, Nutricia, Mead Johnson, Pfizer, Astra Zeneca, and MSD. He has received research funding from Danone and Stallergenes. KA has received speaker’s honorarium from Pfizer, Abbott and Danone. ST declares that he has no competing interests. WS, JB, LG, MM, MT, JN, ADG declares that he has no competing interests.

## Authors’ contributions

GZ made substantial contribution to the conception, design and revising the manuscript. KA is local clinical PI on the study and made substantial contributions to the development of the study design and protocol made substantial contribution to the conception and design of the manuscript. ST devised the original research concept with JH, JB and others and has revised the manuscript critically for important intellectual content. WS has revised the manuscript critically for important intellectual content. JB devised the original research concept with JH, ST and others and has revised the manuscript critically for important intellectual content. MT has contributed to refinement of the study protocol and review of manuscript LG reviewed the epidemiological study design, proposed the statistical analysis plan and contributed to the writing and revision of the paper. MM has contributed to the revision of the paper. JN has contributed to the drafting of the manuscript. ADG contributed to study design and has contributed in drafting and revising the manuscript. JH is lead clinical PI on the study and developed the original research concept with ST. He made substantial intellectual contribution to the manuscript, has been involved in drafting and giving final approval of the version to be published. All authors read and approved the final manuscript.

## Authors’ information

Giovanni Zurzolo is a PhD scholar and is funded by the Victoria University. Professor Katrina J. Allen, paediatric gastroenterologist and is funded by the Viertel Senior Medical Research Followership. Steve Taylor is a Professor at the University of Nebraska-Lincoln and co-Director of Food Allergy Research & Resource Program (FARRP), a food industry-funded consortium with 70 supporting food companies. Wayne Shreffler is the Chief of Pediatric Allergy and Immunology at MGH and Associate Professor of Pediatrics at Harvard Medical School. Joseph Baumert is an Assistant Professor at the University of Nebraska-Lincoln and co-Director of Food Allergy Research & Resource Program (FARRP). A/Prof Mimi Tang is the Director of the Department of Allergy and Immunology, Royal Children’s Hospital, Melbourne. A/Prof Lyle Gurrin is a Senior Lecturer in Biostatistics at The Centre for Molecular, Environmental, Genetic and Analytic (MEGA) Epidemiology. A/Prof Michael Mathai is a Senior Lecturer at The College of Health and Biomedicine at Victoria University. Julie Nordlee work’s at FARRP at the University of Nebraska-Lincoln. Audrey DunnGalvin is a Lecturer in Clinical Psychology in UCC, Cork. Jonathan Hourihane is a Professor of Paediatrics and Child Health in UCC, Cork.

## Supplementary Material

Additional file 1 Food Allergy Quality of Life Questionnaire –Parent Form (0–12 years). Click here for file

Additional file 2 Food Allergy Quality of Life Questionnaire–Child Form (8–12 years). Click here for file

Additional file 3 Food Allergy Quality of Life Questionnaire–Teenager Form (13-18 years). Click here for file

Additional file 4 Peanut single dose study, non-participant questionnaire. Click here for file
