# Supplementary material for: Peanut Allergen Threshold Study (PATS): validation of eliciting doses using a novel single-dose challenge protocol
Source: Allergy Asthma Clin Immunol. 2013 Sep 12;9(1):35. doi: 10.1186/1710-1492-9-35 (PMC3850217; doi:10.1186/1710-1492-9-35)
Supplement: Additional file 1 — Food Allergy Quality of Life Questionnaire –Parent Form (0–12 years). [file 1710-1492-9-35-S1.pdf]

The Royal Children's Hospital Melbourne
50 Flemington Road
Parkville Victoria 3052 Australia
TELEPHONE +61 3 9345 5522
www.rch.org.au

The Royal Children's Hospital Melbourne

FAQLQ-PF

Food Allergy Quality of Life Questionnaire – Parent Form (0-12 years)

Food Allergy Quality of Life Questionnaire-Parent Form
(FAQLQ-PF)
Children aged 0-12 years

Instructions to Parents

- The following are scenarios that parents have told us affect children's quality of life because of food allergy.

- Please indicate how much of an impact each scenario has on **your child's quality of life** by placing a tick or an x in one of the boxes numbered 0-6.

Response Options

0 = not at all
1= a little bit
2 = slightly
3 = moderately
4 = quite a bit
5 = very much
6 = extremely

All information given is completely confidential.
This questionnaire will only be identified by a code number.

There are 4 sections to this questionnaire : A, B, C, and D.

- If your child is aged 0 to 3 years, please answer Section A
- If your child is aged 4 to 6 years, please answer Section A and Section B
- If your child is aged 7 years and over, please answer Section A, Section B, and Section C.

Section D : For all age groups.

SECTION A : For all age groups

**Because of food allergy, my child feels……………**	**0** Not at all	**1**	**2**	**3**	**4**	**5**	**6** Extremely
1 Worried about food	☐	☐	☐	☐	☐	☐	☐
2 Different from other children	☐	☐	☐	☐	☐	☐	☐
3 Frustrated by dietary restrictions	☐	☐	☐	☐	☐	☐	☐
4 Afraid to try unfamiliar foods	☐	☐	☐	☐	☐	☐	☐
5 Concerned that I am worried that he/she will have a reaction to food	☐	☐	☐	☐	☐	☐	☐

**Because of food allergy, my child……………**	**0** Not at all	**1**	**2**	**3**	**4**	**5**	**6** Extremely
6 Experiences physical distress	☐	☐	☐	☐	☐	☐	☐
7 Experiences emotional distress	☐	☐	☐	☐	☐	☐	☐
8 Has a lack of variety in his her diet	☐	☐	☐	☐	☐	☐	☐

**Because of food allergy, my child has been negatively affected by………..**	**0** Not at all	**1**	**2**	**3**	**4**	**5**	**6** Extremely
9 Receiving more attention more attention than other children of his/her age	☐	☐	☐	☐	☐	☐	☐
10 Having to grow up more quickly than other children of his/her age	☐	☐	☐	☐	☐	☐	☐
11 His/her environment being more restricted than other children of his/her age	☐	☐	☐	☐	☐	☐	☐

**Because of food allergy, my child's social environment is restricted because of limitations on……..**	**0** Not at all	**1**	**2**	**3**	**4**	**5**	**6** Extremely
12 Restaurants we can safely go to as a family	☐	☐	☐	☐	☐	☐	☐
13 Holiday destinations we can safely go to as a family	☐	☐	☐	☐	☐	☐	☐

**Because of food allergy, my child's ability to take part has been limited……..**	**0** Not at all	**1**	**2**	**3**	**4**	**5**	**6** Extremely
14 In social activities in other people's houses (*sleepovers, parties, playtime*)	☐	☐	☐	☐	☐	☐	☐

SECTION B : For children aged 4 to 12 years.

**Because of food allergy, my child's ability to take part has been limited……..**	**0** Not at all	**1**	**2**	**3**	**4**	**5**	**6** Extremely
15 In preschool/school events involving food (*class parties/treats/lunchtime*)	☐	☐	☐	☐	☐	☐	☐

**Because of food allergy, my child feels……………**	**0** Not at all	**1**	**2**	**3**	**4**	**5**	**6** Extremely
16 Worried when going to unfamiliar places	☐	☐	☐	☐	☐	☐	☐
17 Concerned that he/she must always be cautious about food	☐	☐	☐	☐	☐	☐	☐
18 'Left out' in activities involving food	☐	☐	☐	☐	☐	☐	☐
19 Upset that family social outings have been restricted by the need to plan ahead.	☐	☐	☐	☐	☐	☐	☐
20 Concerned about accidentally eating an ingredient to which he/she is allergic	☐	☐	☐	☐	☐	☐	☐
21 Worried when eating with unfamiliar adults/children	☐	☐	☐	☐	☐	☐	☐
22 Frustrated by social restrictions	☐	☐	☐	☐	☐	☐	☐

**Because of food allergy, my child……………**	**0** Not at all	**1**	**2**	**3**	**4**	**5**	**6** Extremely
23 Is more worried in general than other children of his/her age	☐	☐	☐	☐	☐	☐	☐
24 Is more cautious in general than other children of his/her age	☐	☐	☐	☐	☐	☐	☐
25 Is not as confident as other children of his/her age in social situations	☐	☐	☐	☐	☐	☐	☐
26 Wishes his/her food allergy would go away	☐	☐	☐	☐	☐	☐	☐

SECTION C : For children aged 7 to 12 years

**Because of food allergy, my child feels……………**	**0** Not at all	**1**	**2**	**3**	**4**	**5**	**6** Extremely
27 Worried about his/her future(opportunities, relationships)	☐	☐	☐	☐	☐	☐	☐
28 Many people do not understand the serious nature of food allergy	☐	☐	☐	☐	☐	☐	☐
29 Concerned by poor labelling on food products	☐	☐	☐	☐	☐	☐	☐
30 Food allergy limits his/her life in general	☐	☐	☐	☐	☐	☐	☐

Thank you for completing the questionnaire. I would be grateful if you would now answer some questions on your child's food allergy.

SECTION D: For all age groups

Part 1 : My child's food allergy.

Q1. What sex are you ? Male ☐ Female ☐

Q2. What sex is your child? Male ☐ Female ☐

Q3. What age is the child with food allergy? Years _______ Months ________

☐ ☐

Q4. What type of food(s) is your child allergic to? *Tick where applicable.*

Peanut	☐	Nut	☐	Milk	☐	Egg	☐
Wheat	☐	Soya	☐	Sesame	☐	Fish	☐
Shellfish	☐	Fruits	☐	Vegetables	☐	Other	☐

Please specify '**Other'**

Q5. After ingesting which food, did your child have his/her most severe reaction?

Q6. Has your child had an anaphylactic reaction? Yes ☐ No ☐

Q7. If 'Yes', how recent was the reaction? *Tick where applicable.*

Very recently ☐

6 to 12 months ago ☐

Approximately 1 yr ago ☐

Approximately 2yrs ago ☐

More than 2 years ago ☐

Q8(a). Has your child been issued with an anapen/epipen? Yes ☐ No ☐

Q8(b). Does the provision of an anapen/epipen cause?

**(1) Reassurance …**	For you ☐	For your child ☐
**(2) Anxiety …**	For you ☐	For your child ☐

Q9. Who diagnosed your child with food allergy? *Tick where applicable*
.
G.P. ☐

Consultant Allergist ☐

Consultant Paediatrician ☐

Dermatologist ☐

Dietician ☐

Alternative Practitioner ☐

Q10. What Symptoms does your child have? *Tick where applicable.*

Itching in the mouth		Throat tightening		Urticaria/Hives	
Itching in the throat		Difficulty swallowing		Skin swelling	
Itching in the ears		Hoarseness		Nausea	
Itching of the lips		Difficulty breathing		Abdominal cramps	
Runny nose		Shortness of breath		Vomiting	
Stuffy nose		Wheeze		Diarrhoea	
Sneeze		Cough		Light-headedness	
Itchy eyes		Itching of the skin		Palpitations	
Tears		Redness of the skin		Inability to stand	
Red eyes		Increase eczema		Loss of consciousness	

Q11. How often does your child meet another child with food allergy?

Never ☐

Rarely ☐

Sometimes ☐

Often ☐

SECTION E: For all age groups

Part 2 : You and your child's worries about food safety

**0 = extremely unlikely**
**1 = very unlikely**
**2 = somewhat unlikely**
**3 = likely**
**4 = quite likely**
**5 = very likely**
**6 = extremely likely**

Please answer the following questions with reference to the 6-point scale on the right

Q1. What chance **do you think** your child has of ….?

	**Question**	**6-point Scale**						
		**0**	**1**	**2**	**3**	**4**	**5**	**6**
**1**	……accidentally ingesting the food to which they are allergic ?							
**2**	……having a severe reaction if food is accidentally ingested ?							
**3**	……dying from his/her food allergy following ingestion in the future ?							
**4**	……effectively treating him/herself, or receiving effective treatment from others (including Epipen administration), if he/she accidentally ingests a food to which he/she is allergic ?							

Q2. What chance **does your child think** he/she has of ……?

	**Question**	**6-point Scale**						
		**0**	**1**	**2**	**3**	**4**	**5**	**6**
**1**	……accidentally ingesting the food to which they are allergic ?							
**2**	……having a severe reaction if food is accidentally ingested ?							
**3**	……dying from his/her food allergy following ingestion in the future ?							
**4**	……effectively treating him/herself, or receiving effective treatment from others (including Epipen administration), if he/she accidentally ingests a food to which he/she is allergic ?							

Q3. How many foods **does your child** have to avoid ?

**0-2**	
**3-6**	
**7-10**	
**10+**	

SECTION F: For all age groups

Part 3: Your concerns as a parent

Q1. How would you describe …

(A) *Your* general health?

- Excellent ☐
- Very Good ☐
- Good ☐
- Fairly Good ☐
- Not So Good ☐
- Poor ☐
- Very Poor ☐

(B) *Your child's* general health?

- Excellent ☐
- Very Good ☐
- Good ☐
- Fairly Good ☐
- Not So Good ☐
- Poor ☐
- Very Poor ☐

Q2. Because of food allergy, how much worry/concern does each of the following cause *you*?

(A) your child's physical health

- None at all ☐
- A little bit ☐
- Some ☐
- Quite a bit ☐
- A lot ☐

(B) your child's emotional well-being

- None at all ☐
- A little bit ☐
- Some ☐
- Quite a bit ☐
- A lot ☐

Q3. What level of stress does your child's food allergy cause …

(A) You?

- None at all ☐
- A little bit ☐
- Some ☐
- Quite a bit ☐
- A lot ☐

(B) Your Partner?

- None at all ☐
- A little bit ☐
- Some ☐
- Quite a bit ☐
- A lot ☐

(C) Your Family?

- None at all ☐
- A little bit ☐
- Some ☐
- Quite a bit ☐
- A lot ☐

Q4. How much has food allergy limited the type of activities…..

(A) you can do as a family ?

- None at all ☐
- A little bit ☐
- Some ☐
- Quite a bit ☐
- A lot ☐

(B) your child can take part in ?

- None at all ☐
- A little bit ☐
- Some ☐
- Quite a bit ☐
- A lot ☐

Thank you for taking the time to complete this questionnaire. Your participation is most appreciated.
