# Supplementary material for: Peanut Allergen Threshold Study (PATS): validation of eliciting doses using a novel single-dose challenge protocol
Source: Allergy Asthma Clin Immunol. 2013 Sep 12;9(1):35. doi: 10.1186/1710-1492-9-35 (PMC3850217; doi:10.1186/1710-1492-9-35)
Supplement: Additional file 2 — Food Allergy Quality of Life Questionnaire–Child Form (8–12 years). [file 1710-1492-9-35-S2.pdf]

The Royal Children's Hospital Melbourne
50 Flemington Road
Parkville Victoria 3052 Australia
TELEPHONE +61 3 9345 5522
www.rch.org.au

The Royal Children's Hospital Melbourne

FAQLQ-CF

Food Allergy Quality of Life Questionnaire – Child Form (8-12 years)

The questions are about the influence of your food allergy on your quality of life. It is important that you fill in the answers yourself. You may ask your parents for help, but they are not allowed to tell you which answer to give. Answer every question by putting an ‘x’ in the proper box. You may choose from the following answers.

☺	☺	☺	😐	☹	☹	☹
not	barely	a little bit	fairly	quite	very	extremely

	**How *troublesome* do you find it, because of your food allergy, that you …**	☺	☺	☺	😐	☹	☹	☹
1	must always watch what you eat?	☐	☐	☐	☐	☐	☐	☐
2	can eat fewer things?	☐	☐	☐	☐	☐	☐	☐
3	are limited in buying things you like?	☐	☐	☐	☐	☐	☐	☐
4	have to read labels?	☐	☐	☐	☐	☐	☐	☐
5	have to refuse food when you do things with others?	☐	☐	☐	☐	☐	☐	☐
6	can less easily stay for a meal with someone?	☐	☐	☐	☐	☐	☐	☐
7	can taste or try fewer things when eating out?	☐	☐	☐	☐	☐	☐	☐
8	have to tell beforehand about what you are not allowed to eat when eating out?	☐	☐	☐	☐	☐	☐	☐
9	have to check yourself whether you can eat something when eating out?	☐	☐	☐	☐	☐	☐	☐
10	hesitate eating certain foods when you don’t know if it is safe?	☐	☐	☐	☐	☐	☐	☐
11	must watch out when touching certain foods?	☐	☐	☐	☐	☐	☐	☐
12	don’t get anything when someone is giving treats at school?	☐	☐	☐	☐	☐	☐	☐

not barely a little bit fairly quite very extremely

**How *troublesome* is it, because of your food allergy, …**							
13 that the ingredients of a food change?	☐	☐	☐	☐	☐	☐	☐
14 that the label states: “May contain (traces of)….”?	☐	☐	☐	☐	☐	☐	☐
15 that you have to explain to people around you that you have a food allergy?	☐	☐	☐	☐	☐	☐	☐
16 that people around you forget that you have a food allergy?	☐	☐	☐	☐	☐	☐	☐
17 that others can eat the food you are allergic to when you do things with other people?	☐	☐	☐	☐	☐	☐	☐
18 that you don’t know how things taste which you can’t eat?	☐	☐	☐	☐	☐	☐	☐

**How *frightened* are you because of your food allergy …**							
19 of an allergic reaction?	☐	☐	☐	☐	☐	☐	☐
20 of eating the wrong food by accident?	☐	☐	☐	☐	☐	☐	☐
21 to eat something you have never eaten before?	☐	☐	☐	☐	☐	☐	☐

**Answer the following questions:**							
22 How *concerned* are you that you will never get rid of your food allergy?	☐	☐	☐	☐	☐	☐	☐
23 How *disappointed* are you when people don’t take your food allergy into account?	☐	☐	☐	☐	☐	☐	☐
24 How *disappointed* do you feel because you have a food allergy?	☐	☐	☐	☐	☐	☐	☐

The following four questions are about the chance that you think you have of something happening to you because of your food allergy. Choose one of the answers. This is followed by two more questions about your food allergy. Answer every question by putting an ‘x’ in the box next to the proper answer.

**0**	**1**	**2**	**3**	**4**	**5**	**6**
**never (0% chance)**	**very small chance**	**small chance**	**fair chance**	**big chance**	**very big chance**	**always (100% chance)**

	**How big do you think the chance is that you …**	**0**	**1**	**2**	**3**	**4**	**5**	**6**
**1**	will accidentally eat something to which you are allergic?	☐	☐	☐	☐	☐	☐	☐
**2**	will have a severe reaction if you accidentally eat something to which you are allergic?	☐	☐	☐	☐	☐	☐	☐
**3**	will die if you accidentally eat something to which you are allergic?	☐	☐	☐	☐	☐	☐	☐
**4**	can **not** do the right things for your allergic reaction should you accidentally eat something to which you are allergic?	☐	☐	☐	☐	☐	☐	☐

**5. How many foods are you unable to eat because of your food allergy?**	**6.** Everyone does things with other people, such as;<br>- playing with friends,<br>- going to a birthday party,<br>- visiting,<br>- staying over with someone for a meal or eating out.<br>**How much does your food allergy affect things you do with others?**
☐ almost none	☐ so little I don’t actually notice it
☐ very few	☐ very little
☐ a few	☐ little
☐ some	☐ moderately
☐ many	☐ a good deal
☐ very many	☐ a great deal
☐ almost all	☐ a very great deal
