# Supplementary material for: Peanut Allergen Threshold Study (PATS): validation of eliciting doses using a novel single-dose challenge protocol
Source: Allergy Asthma Clin Immunol. 2013 Sep 12;9(1):35. doi: 10.1186/1710-1492-9-35 (PMC3850217; doi:10.1186/1710-1492-9-35)
Supplement: Additional file 3 — Food Allergy Quality of Life Questionnaire–Teenager Form (13-18 years). [file 1710-1492-9-35-S3.pdf]

The Royal Children's Hospital Melbourne
50 Flemington Road
Parkville Victoria 3052 Australia
TELEPHONE +61 3 9345 5522
www.rch.org.au

The Royal Children's Hospital Melbourne

FAQLQ-TF

Food Allergy Quality of Life Questionnaire – Teenager Form (13-18 years)

The following questions concern the influence your food allergy has on your quality of life. Answer every question by marking the appropriate box with an 'x'. You may choose from one of the following answers.

0	1	2	3	4	5	6
not	barely	slightly	moderately	quite	very	extremely

	**How *troublesome* do you find it, because of your food allergy, that you …**	**0**	**1**	**2**	**3**	**4**	**5**	**6**
1	must always be alert as to what you are eating?	☐	☐	☐	☐	☐	☐	☐
2	are able to eat fewer products?	☐	☐	☐	☐	☐	☐	☐
3	are limited as to the products you can buy?	☐	☐	☐	☐	☐	☐	☐
4	must read labels?	☐	☐	☐	☐	☐	☐	☐
5	have the feeling that you have less control of what you eat when eating out?	☐	☐	☐	☐	☐	☐	☐
6	are less able to spontaneously accept an invitation to stay for a meal?	☐	☐	☐	☐	☐	☐	☐
7	are less able to taste or try various products when eating out?	☐	☐	☐	☐	☐	☐	☐
8	must check yourself whether you can eat something when eating out?	☐	☐	☐	☐	☐	☐	☐
9	hesitate eating a product when you have doubts about it?	☐	☐	☐	☐	☐	☐	☐
10	must refuse treats at school or work?	☐	☐	☐	☐	☐	☐	☐
11	must be careful about touching certain foods?	☐	☐	☐	☐	☐	☐	☐
12	must carry an epinephrine auto injector (e.g. EpiPen, Twinject, Anapen)? (If you don't have an epinephrine auto injector mark an 'x' here ☐)	☐	☐	☐	☐	☐	☐	☐

0	1	2	3	4	5	6
not	barely	slightly	moderately	quite	very	extremely

**How *troublesome* is it, because of your food allergy, …**	**0**	**1**	**2**	**3**	**4**	**5**	**6**
13 that the ingredients of a food change?	☐	☐	☐	☐	☐	☐	☐
14 that the label states: "May contain (traces of)…."?	☐	☐	☐	☐	☐	☐	☐
15 that the labeling of the bulk packaging (for example box or bag) is different than the individual packages?	☐	☐	☐	☐	☐	☐	☐
16 that you have to explain to people around you that you have a food allergy?	☐	☐	☐	☐	☐	☐	☐
17 that during social activities others can eat the food to which you are allergic?	☐	☐	☐	☐	☐	☐	☐
18 that during social activities your food allergy is not taken into account enough?	☐	☐	☐	☐	☐	☐	☐

**How *frightened* are you because of your food allergy …**	**0**	**1**	**2**	**3**	**4**	**5**	**6**
19 of an allergic reaction?	☐	☐	☐	☐	☐	☐	☐
20 of accidentally eating the wrong food?	☐	☐	☐	☐	☐	☐	☐
21 to eat something you have never eaten before?	☐	☐	☐	☐	☐	☐	☐

**Answer the following questions:**	**0**	**1**	**2**	**3**	**4**	**5**	**6**
22 How *discouraged* do you feel during an allergic reaction?	☐	☐	☐	☐	☐	☐	☐
23 How *disappointed* are you when people don't take your food allergy into account?	☐	☐	☐	☐	☐	☐	☐

The following four questions are about the chance that you think you have of something happening to you because of your food allergy. Choose one of the answers. This is followed by two more questions about your food allergy. Answer every question by putting an ‘x’ in the box next to the proper answer.

0	1	2	3	4	5	6
**never (0% chance)**	**very small chance**	**small chance**	**fair chance**	**great chance**	**very great chance**	**always (100% chance)**

	**How great do you think the chance is that you …**	**0**	**1**	**2**	**3**	**4**	**5**	**6**
**1**	will accidentally eat something to which you are allergic?	☐	☐	☐	☐	☐	☐	☐
**2**	will have a severe reaction if you accidentally eat something to which you are allergic?	☐	☐	☐	☐	☐	☐	☐
**3**	will die if you accidentally eat something to which you are allergic?	☐	☐	☐	☐	☐	☐	☐
**4**	can **not** effectively deal with an allergic reaction should you accidentally eat something to which you are allergic?	☐	☐	☐	☐	☐	☐	☐

**5. How many products must you avoid because of your food allergy?**	**6. How great is the impact of your food allergy on your social life?**
☐ almost none	☐ negligibly small
☐ very few	☐ very small
☐ a few	☐ small
☐ some	☐ moderate
☐ many	☐ great
☐ very many	☐ very great
☐ almost all	☐ extremely great
