# Supplementary material for: Peanut Allergen Threshold Study (PATS): validation of eliciting doses using a novel single-dose challenge protocol
Source: Allergy Asthma Clin Immunol. 2013 Sep 12;9(1):35. doi: 10.1186/1710-1492-9-35 (PMC3850217; doi:10.1186/1710-1492-9-35)
Supplement: Additional file 4 — Peanut single dose study, non-participant questionnaire. [file 1710-1492-9-35-S4.pdf]

The Royal Children's Hospital Melbourne
50 Flemington Road
Parkville Victoria 3052 Australia
TELEPHONE +61 3 9345 5522
www.rch.org.au

The Royal Children's Hospital Melbourne

Additional file 1
Peanut single dose study, non-participant questionnaire

Dear Parent/Guardian,
This questionnaire is voluntary and the information that you provide will be used to help us determine if those that choose to participate are different to those that choose not to.

Name of Child

1) How was your child diagnosed with peanut allergy? ☐
Positive SPT but never ingested ☐
Positive SPT and history of reaction ☐

2) If your child has a history of reaction what is the most severe reaction to peanut ingestion your child has had:

(a) 1 or more of the following: hives, face swelling, vomiting, diarrhoea, eczema flare ☐

(b) Any of the above plus any of one of the following: coughing, wheezing, difficulty breathing, throat or tongue swelling, change in voice, collapse ☐

3) When was your child's last ingestion reaction?
Within the last 1 year ☐
Within the last 5 years ☐
Never ☐

4) How many reactions to peanut has your child had?

5) Are you currently ignoring precautionary labelling when feeding your child?

Yes ☐ No ☐

6) Why have you decided not to participate (tick as many as apply)
Wish to continue to avoid precautionary labelling ☐
Frightened of a serious reaction to the single-dose challenge ☐
Have had anaphylaxis in the past ☐
Don't have time ☐
Other - please specify ☐

I hereby give permission for the Peanut Allergy Threshold Study staff to examine my child's clinical notes and to use peanut allergy-related information from these notes in the study. These details will be anonymised and can only be used in such a way as to not be traceable specifically back to my child.

Signed ____
Relation to Child Mother /father/ legal guardian
Witnessed by ____(Research staff)
